# Acid Stripping after Infection Improves the Detection of Viral HLA Class I Natural Ligands Identified by Mass Spectrometry

**DOI:** 10.3390/ijms221910503

**Published:** 2021-09-29

**Authors:** Elena Lorente, Miguel Marcilla, Patricia G. de la Sota, Adriana Quijada-Freire, Carmen Mir, Daniel López

**Affiliations:** 1 Centro Nacional de Microbiología, Instituto de Salud Carlos III, 28220 Majadahonda, Spain; elorente@isciii.es (E.L.); patric15@ucm.es (P.G.d.l.S.); aquijada@cnb.csic.es (A.Q.-F.); carmen.mir@isciii.es (C.M.); 2 Proteomics Unit, Spanish National Biotechnology Center (CNB-CSIC), 28049 Madrid, Spain; mmarcilla@cnb.csic.es

**Keywords:** HLA class I, immunoproteomics, ligand, peptide, virus

## Abstract

Identification of a natural human leukocyte antigen (HLA) ligandome is a key element to understand the cellular immune response. Advanced high throughput mass spectrometry analyses identify a relevant, but not complete, fraction of the many tens of thousands of self-peptides generated by antigen processing in live cells. In infected cells, in addition to this complex HLA ligandome, a minority of peptides from degradation of the few proteins encoded by the viral genome are also bound to HLA class I molecules. In this study, the standard immunopeptidomics strategy was modified to include the classical acid stripping treatment after virus infection to enrich the HLA ligandome in virus ligands. Complexes of HLA-B*27:05-bound peptide pools were isolated from vaccinia virus (VACV)-infected cells treated with acid stripping after virus infection. The HLA class I ligandome was identified using high throughput mass spectrometry analyses, yielding 37 and 51 natural peptides processed and presented untreated and after acid stripping treatment VACV-infected human cells, respectively. Most of these virus ligands were identified in both conditions, but exclusive VACV ligands detected by mass spectrometry detected on acid stripping treatment doubled the number of those identified in the untreated VACV-infected condition. Theoretical binding affinity prediction of the VACV HLA-B*27:05 ligands and acute antiviral T cell response characterization in the HLA transgenic mice model showed no differences between HLA ligands identified under the two conditions: untreated and under acid stripping condition. These findings indicated that acid stripping treatment could be useful to identify HLA class I ligands from virus-infected cells.

## 1. Introduction

Proteasomes and other cytosolic peptidases degrade self and pathogenic proteins, generating an extremely varied pool of peptides, both in sequence and in length. Some of these degradation products are translocated to the endoplasmic reticulum (ER) lumen by the transporter associated with antigen-processing (TAP) molecules [1]. The peptides with the correct length (8 to 12 residues) and strong interactions with specific residues of the antigen recognition site of the HLA class I molecule [2], usually at position 2 (P2) as an anchor residue motif and auxiliary amino acids at the C-terminus [3,4], can stabilize their binding to HLA class I molecules. Amino-terminally extended precursors can be also utilized for antigen presentation, generally (but not exclusively) after precursor trimming by ER-resident aminopeptidase activities [5,6,7]. The specific interaction of peptide with the HLA class I molecule in the ER stabilizes the nascent trimolecular peptide-HLA-β_2_-microglobulin complexes and allows for their subsequent export to the cell membrane, where they can interact with the CD8 receptor [8]. The recognition of foreign or self-peptide ligands can lead to the beneficial killing of pathogen-infected cells or, instead, to initiate an autoimmune damage, respectively.

Advanced high throughput mass spectrometry has contributed significantly to identify virus-derived natural ligands presented by HLA molecules, a key element to understand the cellular antiviral immune responses [9]. These massive analyses can resolve the sequence of several thousand HLA-bound peptides for each experiment. However, the hundreds of thousands of HLA class I molecules expressed on the surface of a cell [10] can bind many tens of thousands of peptides in different amounts. In infected cells, additional to this complex self HLA ligandome, the peptides generated by the antigen processing of the dozens or few hundred of viral proteins encoded by the pathogen genome are also presented in the cell surface bound to the HLA class I molecules. In the determination of targets of cellular immune response, these self-peptides are not only irrelevant but also obstruct the identification of pathogen-derived ligands due to their extreme diversity and abundance. Thus, methods that increase the virus/self ratio of the HLA class I ligands analyzed by mass spectrometry would be highly desirable. In this context, the depletion of cell surface HLA class I molecules after virus infection by the classical acid stripping treatment [11] carried out in this study improved the identification of vaccinia virus (VACV) ligands detected by mass spectrometry.

## 2. Results

### 2.1. Physiological Processing Generated Different Viral HLA-B*27:05 Ligands in Human VACV-Infected Cells

The HLA-B*27:05-bound peptide pools were isolated from large numbers of acid-untreated and either uninfected or VACV-infected cells. These peptide mixtures were subsequently separated by reversed-phase HPLC and were analyzed by LC-MS/MS. By means of bioinformatics tools, different fragmentation spectra detected in the VACV-infected HLA-bound peptide pools (but absent from both control uninfected pools), corresponded, with high confidence, to VACV-derived peptides (Table 1). Additionally, a human proteome database search failed to identify any of these spectra as human protein fragments, supporting the viral origin of these ligands. Eighteen different VACV proteins (3BHS, A31, B18, C10, D2, E8, F7, F11, F12, F17, I1, K1, MCE, RP19, RP22, RP35, VGF, and VLTF1) displayed individual ligands bound to HLA-B*27:05 molecules (Table 1). In addition, another five VACV proteins (B25, C13, H3, RP132, and VITF3) yielded two HLA-B*27:05 ligands each (Table 1). Moreover, three different viral ligands from B1 protein were identified bound to HLA-B*27:05 molecules (Table 1). Finally, the VACV E5 protein also generated six HLA-B*27:05 ligands. (Table 1). Therefore, these results indicate that a total of 37 HLA-B*27:05 ligands from 28 different VACV proteins were processed and presented in VACV-infected human cells.

### 2.2. Diverse Viral HLA-B*27:05 Ligands Were Physiological Generated in Human VACV-Infected Cells Treated with Acid Stripping

Similar to previous conditions, the HLA-B*27:05-bound peptide pools were isolated from either uninfected or VACV-infected cells treated with acid stripping after virus infection. Different fragmentation spectra present in the HLA-B*27:05-bound peptide pools, but absent in the uninfected pools, were also matched to VACV-derived peptide sequences (Table 1). As previously, a human proteome database search failed to identify any of these spectra as human protein fragments, supporting the viral origin of these ligands. Twenty two different VACV proteins (3BHS, A27, A32, A37, A52, B18, C13, D2, D9, D13, E6, F7, F12, G7, H3, H5, K1, PAP1, RP19, RP22, RP35, and SPI2) displayed individual ligands bound to HLA-B*27:05 molecules (Table 1). In addition, the B25, C10, DUT, and F17 proteins yielded two HLA-B*27:05 ligands each (Table 1). Moreover, three different viral ligands from EFT1, I1, and RP132 proteins were identified bound to HLA-B*27:05 molecules (Table 1). Finally, VACV B1, and E5 proteins were the main contributors to antigen processing and presentation with 4 and 7 ligands bound to HLA-B*27:05 molecules (Table 1). Therefore, the immunoproteomic analysis identified a total of 51 HLA-B*27:05 ligands from 32 different VACV proteins found in VACV-infected human cells after acid stripping of infected cells.

### 2.3. Most of Viral HLA-B*27:05 Ligands Were Independent of Acid Stripping Treatment

**Figure 1:**
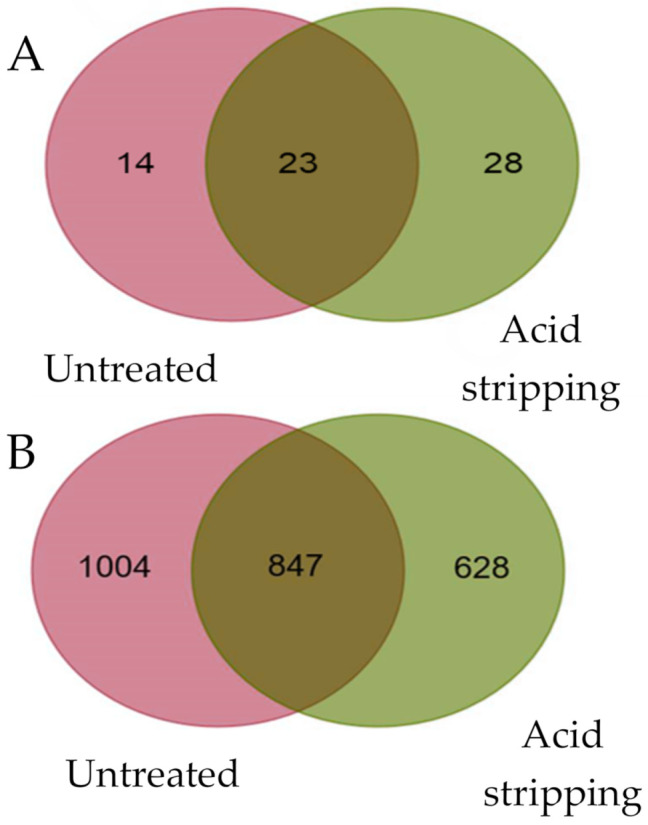
Overlap of HLA-B*27:05 viral and cell ligands from untreated and acid stripping condition VACV-infected cells. Venn diagram showing number de viral (**A**) or cell (**B**) ligands identified exclusively in untreated virus-infected cells (pink), acid stripping condition (green) VACV-infected cells and overlapping peptides between both conditions.

A total of 37 and 51 VACV HLA-B*27:05 ligands were identified in untreated cells and after acid stripping VACV-infected human cells, respectively (Table 1). Twenty three of these virus ligands were identified in both conditions; 14 peptides were exclusively identified in untreated VACV-infected human cells and another 28 ligands were bound to HLA-B*27:05 in VACV-infected human cells after acid stripping (Table 1 and Figure 1A). Thus, a relevant fraction of VACV ligands detected by mass spectrometry was identified in the acid stripping treatment and this condition allowed the identification of twofold more exclusive viral ligands that the untreated VACV-infected condition. In contrast to viral ligands, about 20% more self-ligands were identified in untreated VACV-infected conditions versus the virus-infected cells after acid stripping treatment (Figure 1B).

### 2.4. Theoretical Binding Affinity of the VACV HLA-B*27:05 Ligands

**Figure 2:**
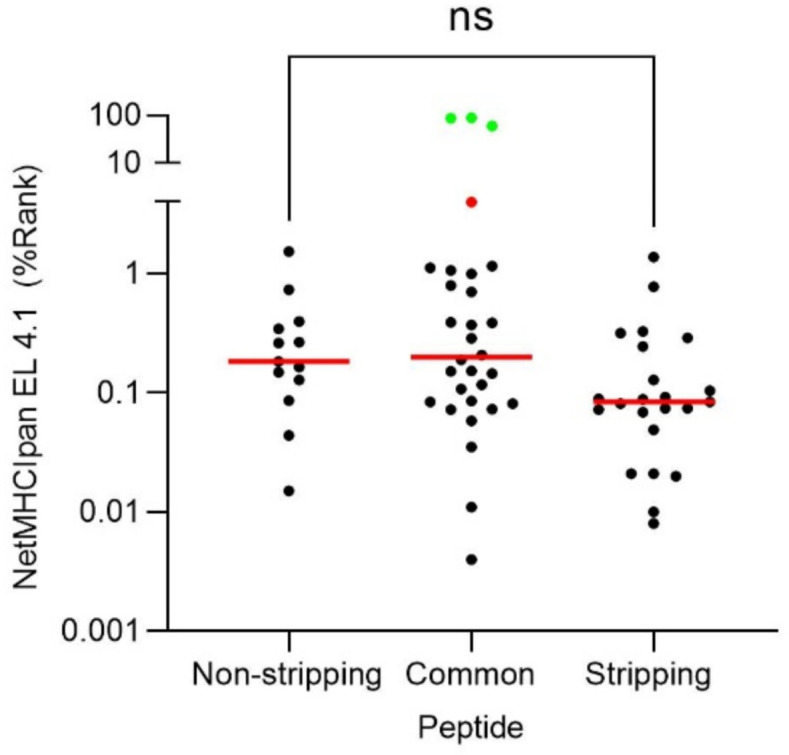
Theoretical binding affinity of the VACV HLA-B*27:05 ligands identified by mass spectrometry. The theoretical binding affinity to the HLA-B*27:05 class I molecule of the different VACV ligands (classified by exclusives for untreated or acid stripping conditions, as well as peptides identified in these two conditions or common peptides) was calculated with the NetMHCIpan EL 4.1 server. The threshold for strong binding peptides (marked as black dots) was 2 of the percentile rank. One peptide (marked as red dot) ranked as weak binding. In addition, another 3 peptides (marked as green dots) showed no theoretical affinity for HLA-B*27:05.

Prediction of HLA peptide binding by bioinformatics software is routinely utilized to select potential candidates for viral ligands and/or to determinate the binding affinity of experimentally detected HLA ligands. Therefore, prediction of the peptide binding of the 65 VACV ligands to the HLA-B*27:05 class I molecule was analyzed using the NetMHCIpan neural network-based alignment method. The 13 peptides exclusively associated with untreated VACV-infected cells, the 23 ligands only identified with the acid stripping treatment, and 25 of the 29 virus peptides identified in both conditions were predicted as high affinity ligands for HLA-B*27:05 binding (black dots, Figure 2). The SQFDDKGNTAL peptide was predicted by the bioinformatics tool as a low affinity ligand (red dot, Figure 2), while peptides AANRDNVASRLLN, FAANRDNVASRLLN, and PVIDRLPSETFPNVH were not predicted as HLA-B*27:05 ligands (green dots, Figure 2). Additionally, no statistical differences were observed between HLA ligands identified under the two conditions: untreated and in acid stripping condition (Figure 2).

### 2.5. Immunogenicity of VACV-Derived Peptides Obtained after Acid Stripping in HLA-B*27:05 Transgenic Mice

**Figure 3:**
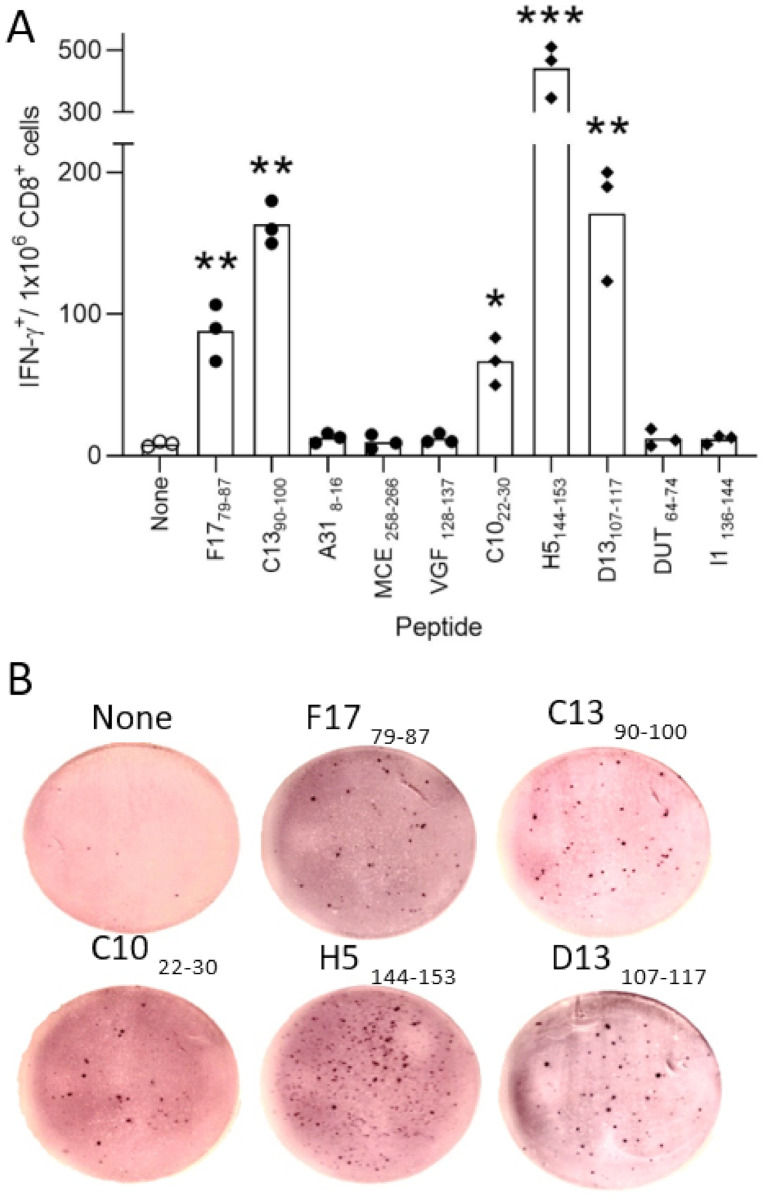
The immunogenicity of VACV-derived HLA-B*27:05-restricted peptides in the HLA class I transgenic mice. (**A**) HLA-B*27:05 target cells that were pre-pulsed with the indicated VACV-synthetic peptides and were analyzed by ELISPOT for CD8^+^ T cell activation with VACV-specific splenocytes obtained from HLA-B*27:05 transgenic mice immunized for 7 days (acute response) post VACV infection. The results are calculated as the mean of three independent experiments ±SD. Significant *p* values (* < 0.05; ** < 0.01; and *** < 0.05) of ligands exclusively identified in untreated (closed circles) or after acid stripping condition (closed diamonds) versus negative control (open circles) is indicated. A representative experiment is depicted in (**B**).

Although the VACV ligands exclusively associated with the acid stripping condition showed similar characteristics of sequence (Table 1) and theoretical binding affinity (Figure 2) to the other HLA-B*27:05 ligands identified in the current study, it could be that these ligands were only expressed after removing cell peptides previously bound to the HLA-B*27:05 class I molecules. To analyze this point, HLA-B*27:05 transgenic mice were infected with VACV. Later, a physiological measurement of the functional ex vivo activity of T cells against different HLA-B*27:05 viral ligands (5 exclusively identified of untreated and other 5 after acid stripping condition VACV-infected cells, underlined in Table 1) identified using mass spectrometry was carried out. Spleen cells that specifically recognized target cells that were pulsed with F17_79–87_ (MRTDMLQNM) and C13_90–100_ (SRFTIGRALFK) peptides (but not the other 3) from an untreated condition were simultaneously recognized as part of the acute response to VACV (Figure 3). The C10_22–30_ (IRNDIRELF) and D13_107–117_ (GRFGYVPYVGY) peptides, exclusively identified after acid stripping treatment, displayed IFN-γ^+^ responses of the same order as F17_79–87_ and C13_90–100_ peptides, respectively (Figure 3). In addition, the H5_144–153_ (ARSDLSDLKV) peptide, also associated to HLA-B*27:05 only after acid stripping treatment, was the epitope and was more immunogenic in the HLA-B*27:05 transgenic mice model (Figure 3). The other two peptides from acid stripping condition were not recognized by T cells. These data indicate that VACV ligands, identified after acid stripping condition, are also physiologically presented in the absence of this treatment, generating potent T cell responses in HLA-B*27:05 transgenic mice.

## 3. Discussion

Classically, the identification of virus-derived ligands presented by HLA molecules has been addressed using partially overlapping synthetic peptides covering the fractional or full sequence of known or hypothesized antigenic proteins, or even the whole proteome when the virus is small enough [12]. The development of high throughput mass spectrometry has contributed to a better understanding of the cellular antiviral immune response with two main advantages over this previous experimental approach. First, the mass spectrometry analyses allow the unbiased characterization of natural ligands bound to HLA molecules in the surface of the infected cells and thus exposed to T cell activity. Second, for large-genome viruses, such as the vaccinia virus (VACV), which encodes complex proteomes of more than 200 proteins, classical overlapping synthetic peptide analyses are both very expensive and technically unfeasible in practice. However, in the mass spectrometry approach there is a problem. In the surface of the infected cell, together with occasional viral ligands, tens or even hundreds of thousands of peptides of cellular origin are also being presented by HLA class I molecules. This self-derived HLA peptidome is so abundant that even using the most modern mass spectrometers cannot be fully analyzed and thus, becomes “noise” that hinders the identification of the additional minority fraction of viral peptides generated in the infection. The acid stripping treatment removes all HLA-bound cell peptides on the cell surface [11]. In infected cells, only the peptides generated by proteolytic degradation of the fraction of newly synthesized viral or self-proteins whose sequence or folding are in some way defective (defective ribosomal products; DRiP) will be translocated to Endoplasmic Reticulum to be complexed with HLA class I molecules. Thus, this relative enrichment in pathogen ligands in addition to the viral hijacking of cell metabolism [13] allow the identification of additional VACV ligands bound to HLA as we demonstrated in the current study.

Although most VACV HLA-B*27:05 ligands were identified in both conditions, a significant fraction of viral peptides was exclusive, either in the untreated or the acid stripping treatment. The self-derived HLA peptidome from both conditions is different since the stripping acid treatment causes strong cellular stress to replace all lost surface proteins, modifying the normal balance of protein synthesis. In addition, the mass spectrometers only resolve the most abundant species in each point of the HPLC gradient and thus, the relative abundance of viral peptides versus self-peptides could explain the differences in the identification of virus ligands. Collectively, the data of our study indicate that, far from replacing the established protocol (since a similar number of VACV ligands is obtained to those published in previous studies [14,15,16,17]), the acid stripping treatment becomes a complementary method to increase the identification of viral ligands that, as experiments in the transgenic mice model have shown, are similarly antigenic to those obtained without the aforementioned treatment. Moreover, a similar number of mass spectrometry ligands identified untreated and after acid stripping condition VACV-infected cells were not recognized in the HLA-B*2705 transgenic mice. This absence of recognition is probably due to the 65 million years of evolutionary divergence between mice and humans, with accumulation of multiple differences in immune system development, activation, and response, in both innate and adaptive immunity [18]. These differences including relevant proteins involved in the antigen processing and presentation pathway as TAP [19] and endoplasmic reticulum-resident aminopeptidases [6,7].

An interesting fact, not strictly related to the objective of this study, was that four viral ligands (identified both in untreated and acid stripping VACV-infected cells) showed weak or no theoretical binding affinity for the HLA-B*27:05 class I molecule. The first was the SQFDDKGNTAL peptide, which was predicted by the bioinformatics tool as a low affinity ligand. This 11-mer peptide had Gln at P2 residue, an anchor motif shared with 3% of natural HLA-B*27:05 ligands [20,21,22]. The HLA-B*27:05 peptides with Gln as an alternative anchor motif presented experimental affinity values in the range commonly observed among natural ligands with the classical Arg at P2 anchor motif for HLA-B*27:05 binding [22]. In addition, AANRDNVASRLLN, FAANRDNVASRLLN, and PVIDRLPSETFPNVH peptides were not predicted as HLA-B*27:05 ligands by the bioinformatics tool. Although they seem to have no anchor motifs, AANRDNVASRLLN and FAANRDNVASRLLN ligands are N-extended peptides of the core NRDNVASRLLN, an 11-mer with the canonical anchor motif for HLA-B*27:05 binding. Similarly, PVIDRLPSETFPNVH peptide is the N-extension of the other canonical DRLPSETFPNVH peptide. The most likely explanation is that these peptides bind to HLA-B*27:05 with the canonical anchor motif at P2 in an N-extended conformation as others previously identified (SYFPEITHI database: http://www.syfpeithi.de (accessed on May, 2021) [4]). These N-extended peptides can show a similar HLA binding affinity to short canonical peptides and, when they have a viral origin, could be highly antigenic and similarly abundant to minimal length epitopes [23]. Thus, it would be recommended to improve the performance of the HLA binding predictors with these non-canonical sequences obtained experimentally by mass spectrometry analysis.

## 4. Materials and Methods

### 4.1. Cells, Antibodies and Synthetic Peptides

B27-C1R is an HLA-B*27:05 transfectant [24] of the human lymphoid cell line HMy2.C1R. Cells were cultured in RPMI 1640 with 10% fetal bovine serum. The monoclonal antibody (mAb) used in this study was W6/32 (specific for a monomorphic HLA class I) [25]. Peptides were synthesized by ProteoGenix (Schiltigheim, France).

### 4.2. Mice

HLA-B*2705 [26] transgenic mice were bred in our animal facilities in strict accordance with the recommendations of the Guide for the Care and Use of Laboratory Animals of the Spanish “Comisión Nacional de Bioseguridad” of the “Ministerio de Medio Ambiente y Medio Rural y Marino” (accreditation number 28079-34A). The protocol was approved by the Committee on Animal Experiment Ethics of the Institute of Health “Carlos III” (Permit Number: PI-283). All of the procedures were performed under sodium pentobarbital anesthesia, and all efforts were made to minimize suffering.

### 4.3. Acid Stripping and HLA Class I Re-Expression

Uninfected or VACV-infected cells after 2 h of infection (to allow entry of the virus) at moi 10 were washed with RPMI in the absence of serum and incubated for 90 s with ice-cool acid-stripping medium (0.3 M glycine-HCl and 1% BSA in water, pH 2.4) as previously reported [27]. Culture medium was added to neutralize the pH. Cells were washed three times, resuspended in culture medium, and incubated at 37 °C overnight to allow normal viral cycle and HLA class I re-expression.

### 4.4. HLA Class I-Bound Peptide Isolation

HLA-bound peptides were isolated from three independent biological replicates of 1 × 10^9^ cells from uninfected or VACV-infected cells, treated or not with acid stripping after virus infection as previously described [28]. The VACV Western Reserve strain was utilized to infect 1 × 10^9^ B27-C1R cells at a multiplicity of infection of 10 plaque-forming units/cell in 100 mL for 2 h at 37 °C, and then cells were washed with PBS, as previously described [28]. These conditions were previously determined as the optimal to obtain infection of all cells without impairing the cell viability. Next, the cells were cultured for 4 h at 37 °C and stained with the Omnitope antiserum-FITC that recognizes VACV virions (ViroStat Inc., Westbrook, ME, USA). Samples were analyzed measuring fluorescence intensity by flow cytometry to confirm VACV infection (1220 ± 97 mean fluorescence intensity in VACV-infected cells versus 4222 ± 38 in non-infected cells stained with the anti-VACV antiserum). Cells were lysed in 1% Igepal CA-630 (Merck KGaA, Darmstadt, Germany), 20 mM Tris/HCl buffer, and 150 mM NaCl, pH 7.5 in the presence of the Protease Inhibitor Cocktail (Merck KGaA, Darmstadt, Germany). After centrifugation, the supernatants were passed first through a control precolumn containing CNBr-activated Sepharose 4B (GE Healthcare, Buckinghamshire, UK) to remove non-specific proteins and peptides. The HLA-B*27:05/peptide complexes were isolated via affinity chromatography from the soluble cell extract fraction with the W6/32 mAb. The HLA-bound peptides were eluted with 1% aqueous trifluoroacetic acid (TFA), separated from the HLA molecules, and concentrated by ultra-filtration with a Vivaspin 2 filter, 5000 MWCO HY (Sartorius Stedim Biotech, Goettingen, Germany), as previously described [29].

### 4.5. LC-MS/MS Analysis

Peptide samples were reconstituted in 6 µL of 0.2% formic acid. 5 µL of each fraction were analyzed by LC-MS/MS in a nano-LC Ultra HPLC (Eksigent, SCIEX, Framingham, MA, USA) coupled online with a 5600 triple TOF mass spectrometer (Sciex) through a Nanospray 3 ion source (SCIEX). The ion source was equipped with a PicoTip emitter (10 μm × 12 cm, New Objective, Woburn, MA, USA). The HPLC setup included an Acclaim PepMapTM 100 trapping column (10μm × 2cm, Thermo Fisher Scientific, Waltham, MA, USA) and a C18 Peptide BEH column (130Å, 1,7μm × 150mm, Waters. Milford, MA, USA). Solvent A and B were 0.1% formic acid in water and acetonitrile, respectively. Peptides were separated at a flow-rate of 250 nL/min at 50 °C under gradient elution conditions as follows: a linear increase from 5% to 30% B in 180 min, a linear increase to 60% B in 20 min, a linear increase to 90% B in 10 min, 90% B for 10 min, a linear decrease to 5% B in 5 min and 5% B for 20 min. Total run time was 250 min. Each acquisition cycle included a survey scan of 250 ms between 350 and 1250 m/z units and a maximum of 25 MS2 spectra scanning between 100 and 1500 m/z units with an accumulation time of 100 ms. The HPLC and the mass spectrometer were respectively controlled with the Eksigent Control (version 3.12, Eksigent) and the Analyst TF software (version 1.7, Eksigent).

### 4.6. MS/MS Ion Search and Peptide Identification

Raw MS/MS data were converted to mgf files with PeakView 1.2 (SCIEX) and searched with Peaks Studio X+”. (Bioinformatics Solutions). The database (42,651 entries) contained the Human Reference Proteome and the Vaccinia (Western Reserve) Proteome downloaded from Uniprot in July 2019. The following parameters were used: no enzyme, MS tolerance of 25 ppm, MS/MS tolerance of 0.05 and oxidation of M and pyroglutamic acid formation from Q and E as variable modifications. Identifications were filtered at a FDR > 1% at the peptide level. Similar amounts of uninfected human cells were used as a negative control to discriminate viral and cellular peptides (included in proteome databases as well as unknown peptides, whose parental proteins may not be included in current human databases) and to exclude erroneous assignments of viral peptides.

### 4.7. In Silico Binding Prediction of HLA-B*27:05 Class I Ligands

The predicted binding of each peptide to HLA-B*27:05 class I molecule was calculated using the artificial neural network-based alignment method NetMHCIpan EL4.1 (available in http://www.cbs.dtu.dk/services/NetMHCpan/ (accessed on May, 2021)). Strong and weak theoretical affinity of ligands to HLA-B*27:05 corresponding to 1 and 5 percentile ranks were considered.

### 4.8. IFN-γ-Secreting CD8^+^ Cell Detection by ELISPOT

ELISPOT assays were performed as previously described [30] to detect antigen-specific CD8^+^ T cell activation. Briefly, purified rat anti-mouse IFN-γ antibody (clone R4-6A2, BD Pharmingen, San Diego, CA, USA) was coated on 96-well MultiScreen HTS HA plates (Millipore, Billerica, MA, USA). The plates were incubated overnight at room temperature and were blocked with medium that was supplemented with 10% fetal bovine serum for 2 h at 37 °C. Duplicate cultures of erythrocyte-depleted spleen cells were prepared from HLA class I-transgenic mice at 7 days (acute response) post intraperitoneal infection with 1 × 10^6^ pfu of VACV at different dilutions with 10^−5^ M peptide. The plates were incubated overnight at 37 °C in a 5% CO_2_ atmosphere and were then washed with PBS-T (PBS 0.05% Tween-20). The plate wells were incubated for 2 h at room temperature with biotinylated anti-mouse IFN-γ mAb clone XMG1.2 (BD Pharmingen), washed with PBS-T, and incubated for 1 h at room temperature with alkaline phosphatase-conjugated streptavidin (Sigma, St. Louis, MO, USA). The plates were additionally washed before adding the BCIP/NBT liquid substrate (Sigma, St. Louis, MO, USA). To enumerate the IFNγ responses, spots were counted and wells were photographed using a Leica EZ4 HD stereo microscope and LAS EZ software (Leica Microsystems, Germany). Additionally, the percentage of CD8^+^ cells was determined after staining spleen cells with FITC-conjugated anti-mouse CD8 antibody (clone KT15, Proimmune, England, UK). Events were acquired on a FACSCanto flow cytometer (BD Biosciences, San Jose, CA, USA) and analyzed using BD FACSDiva software, version 6 (BD Bioscience). To analyze the statistical significance of the assays, a non-parametric Mann–Whitney U test was used. *p* values < 0.05 were considered to be statistically significant.

## Figures and Tables

**Table 1:** Summary of the HLA-B*27:05 ligands detected by MS/MS analysis in VACV-infected cells.

Peptide	Experimental Mass	Length	Protein	Position	Accession	Untreated Cells ^a^	Stripping Condition ^a^
GRYIVKLLI	1073.696	9	3BHS	14–22	P26670	1	1
KRNDEVLFR	1175.6411	9	A27	99–107	P11258	ND	2
LRFLEKTSF ^b^	1139.6338	9	A31	8–16	P24760	2	ND
YRHVPILGR	1109.6458	9	A32	206–214	P68615	ND	2
GRIDNINMSI	1147.5656	10	A37	254–263	P24762	ND	2
GRLFNEIKKF	1250.7135	10	A52	77–86	Q01220	ND	2
YRFLVINR	1079.624	8	B1	103–110	P16913	1	1
YRFLVINRL	1192.708	9	B1	103–111	P16913	1	1
RRGDLETLGY	1178.6044	10	B1	210–219	P16913	ND	2
IRWLGGILPWTK	1438.8448	12	B1	229–240	P16913	2	2
VRIKNDIVVSR	1297.783	11	B18	232–242	P25213	2	3
TRFNPSVLK	1060.6029	9	B25	111–119	Q805H0	3	3
TRFNPSVLKILLH	1536.9139	13	B25	111–123	Q805H0	3	3
IRNDIRELF	1174.6458	9	C10	22–30	P03296	ND	2
TRFYFNMPK	1202.5906	9	C10	323–331	P03296	3	3
SRFTIGRALF	1166.656	10	C13	90–99	P17365	1	2
SRFTIGRALFK	1294.751	11	C13	90–100	P17365	2	ND
ARFDNKSIYR	1268.6625	10	D2	63–72	P04300	ND	3
ARFDNKSIYRI	1381.7466	11	D2	63–73	P04300	1	ND
FRVSTKLLRF	1265.7607	10	D9	69–78	P04311	ND	2
GRFGYVPYVGY	1276.624	11	D13	107–117	P68440	ND	2
GRIAPRSGL	925.5457	9	DUT	64–72	P17374	ND	2
GRIAPRSGLSL	1125.6617	11	DUT	64–74	P17374	ND	2
SRNPSKMVY	1096.5336	9	E5	130–138	P21606	ND	3
IRILVEERF	1173.6869	9	E5	179–187	P21606	3	3
IRILVEERFY	1336.7502	10	E5	179–188	P21606	3	3
WRIIGTQVDK	1214.6771	10	E5	197–206	P21606	3	3
ARYNLKPMYR	1310.6918	10	E5	219–228	P21606	2	2
YRYDDDVENGF	1391.563	11	E5	274–284	P21606	3	3
YRYDDDVENGFIGL	1674.7526	14	E5	274–287	P21606	3	2
KRFDELDINNSY	1512.7208	12	E6	372–383	P21607	ND	2
SRGLSRPLM	1015.5597	9	E8	27–35	P23372	2	ND
VRTIIDENR	1114.6095	9	ETF1	31–39	P04308	ND	2
SRFKKVYIL	1152.7019	9	ETF1	63–71	P04308	ND	1
GRSIRKFSY	1112.609	9	ETF1	453–461	P04308	ND	2
YRQQLELAY	1182.6033	9	F11	247–255	Q80HX7	2	ND
TRTIILVGY	1034.6124	9	F12	275–283	Q80HX6	3	3
MRTDMLQNM	1138.4934	9	F17	79–87	P07396	2	ND
AANRDNVASRLLN	1412.7484	13	F17	89–101	P07396	ND	2
FAANRDNVASRLLN	1559.8168	14	F17	88–101	P07396	ND	1
ARVEFGPLYM	1181.5903	10	F7	57–66	P24359	2	ND
YRHTIESVYF	1313.6404	10	F7	83–92	P24359	ND	3
KRFTHTTAFF	1254.6509	10	G7	99–108	P68716	ND	3
PVIDRLPSETFPNVH	1719.8944	15	H3	12–26	P07240	3	ND
ARIENEMKINR	1372.7245	11	H3	217–227	P07240	2	ND
KRYPGVMYAF	1230.6219	10	H3	266–275	P07240	ND	2
ARSDLSDLKV	1102.5981	10	H5	144–153	P07242	ND	2
VRIPVDLVK	1037.6597	9	I1	51–59	P16714	2	3
KRSATQFNF	1097.5618	9	I1	136–144	P16714	ND	3
TRLYDYFTR	1233.6141	9	I1	210–218	P16714	ND	3
SQFDDKGNTAL	1194.5516	11	K1	88–98	P04297	1	1
VRNKVVVNF	1073.6345	9	MCE	258–266	P07617	2	ND
GRVPSVNEY	1019.5036	9	PAP1	23–31	P23371	ND	2
ARDEPVFVK	1059.5713	9	RAP94	292–300	P68438	ND	3
GRLPLVSEF	1016.5654	9	RP19	96–104	P68611	3	3
ARDPYAVINR	1173.6254	10	RP22	20–29	P68609	1	3
HRFDMTKVDVELFIK	1876.9869	15	RP35	275–289	P24757	1	1
SRVSLEFIR	1105.6244	9	RP132	241–249	Q76ZP7	2	1
SRVSLEFIRR	1261.7255	10	RP132	241–250	Q76ZP7	ND	2
KRITESITDF	1208.64	10	RP132	272–281	Q76ZP7	1	2
GRYSAVFKDSFLR	1544.8099	13	SPI2	70–82	P15059	ND	2
RRTKLPIQDM	1256.7023	10	VGF	128–137	P01136	2	ND
ARTIFNFHLI	1230.6873	10	VITF3	16–25	Q80HV2	3	ND
SRTVEIFER	1135.5985	9	VITF3	106–114	Q80HV2	3	ND
IRIKIDKLR	1153.7659	9	VLTF1	18–26	P68613	1	ND

^a^ Number of mass spectrometry runs from untreated or acid stripping conditions in which each peptide was detected. ND: not detected. ^b^ Underlined indicates peptides analyzed by ELISPOT.

## Data Availability

Data are available via ProteomeXchange with identifier PXD027859.
